# Telomerase: Role in Health and Aging

**DOI:** 10.3390/biomedicines10112957

**Published:** 2022-11-17

**Authors:** Yegor E. Yegorov

**Affiliations:** Engelhardt Institute of Molecular Biology, Russian Academy of Sciences, 119991 Moscow, Russia; yegorov@eimb.ru

We would like to introduce the new volume: “Telomerase and Telomeres: Its Role in Health and Aging 2.0”. The first volume has successfully collected a number of articles and reviews that have received a positive response from its audience. A summary of the first volume can be accessed below. My heartfelt thanks to all the authors and editorial staff for their contributions. We hope that the second volume will also be very successful.

Since our first announcement, an interesting development has occurred in the field of telomere biology. In addition to the two well-known ways of telomere maintenance (telomerase-dependent and ALT), a mode of the direct transmission of telomeres from cell to cell has been presented in the literature [1]. This pathway, and most importantly its possible application in medicine, provides novel opportunities to combat the aging of the immune system, increase the effectiveness of vaccinations (e.g., against COVID-19), and, in the future, develop new cell-rejuvenation technologies, particularly for vascular wall cells. We hope that this type of research will be continued in the future studies. 

At present, it is well-known that not only telomeric DNA, but also hTERT mRNA, can be transferred from cell to cell [2]. In a new publication, the authors attempted to prove that the exosomal transfer of hTERT mRNA from cancer cells to fibroblasts is not only responsible for the appearance of telomerase activity, but also for the phenotypic occurring changes in the latter towards their transformation to CAF (cancer-associated fibroblasts). When telomerase activity is blocked in such experiments, most of the changes to the fibroblasts did not occur [3]. 

A simplified scheme of telomerase involvement in the processes leading to aging or contributing to homeostasis maintenance is presented in Figure 1. Intercellular telomere transfer, as well as the telomerase function itself, is involved in the overall scheme.

The experimental articles and reviews presented in the first volume do not address the scheme presented in its entirety, although they focus on various aspects of telomerase activity regulation, diagnostics, cancer, alternative splicing, cell immortalization, telomerase function in different tissues, mutagenesis issues, and aging problems.

A recent review [4] considered the noncanonical role of the telomerase protein TERT in postmitotic neurons. It is possible that TERT attenuates the effects of toxic proteins involved in the pathogenesis of common neurodegenerative disorders, such as Alzheimer’s and Parkinson’s diseases.

Another recently published review [5] focused on the alternative splicing of hTERT. Since hTERT is the major subunit that is critical for telomerase function, its expression and activity are tightly regulated at many levels. The regulation of hTERT functions by the alternative splicing of its pre-mRNA, which is crucial because even small quantities of hTERT may result in significant cellular consequences. To date, more than twenty splice variants of hTERT have been described in the literature; however, only full-length hTERT is catalytically active. Two splice variants comprise major parts of total hTERT mRNA. The deletion of 36 nucleotides in exon 6 (α variant) leads to the disappearance of a part of the reverse transcriptase domain in the hTERT protein and results in the loss of catalytic activity. The deletion of 182 nucleotides of exons 7 and 8 (β variant) leads to the shift of the reading frame and results in the formation of a premature stop codon in exon 10 and the synthesis of a truncated form of the hTERT protein. This hTERT splice variant acts as a dominant negative.

Several factors, most of which are common splicing regulatory (SR) proteins, were identified as potent regulators of hTERT alternative splicing. However, NOVA1 and Brm were shown to interact with SR proteins and affect alternative splicing. Endonuclease G (EndoG) has a role in telomerase suppression in genotoxic conditions. This nuclease produces antisense oligonucleotide base pairing to sensitive sites of hTERT pre-mRNA and induces a β-splice variant.

An experimental paper [6] showed that ectopic hTERT expression in natural killer (NK) cells enhances their activation and proliferation, extends the in vitro life span, and can be a useful tool in developing NK-based cancer cell therapies. At the same time, ectopic hTERT expression does not lead to the immortalization of NK cells. Perhaps we do not yet know sufficiently well enough the cultivation conditions under which hTERT expression would result in the immortalization of NK cells. On the other hand, the limited proliferation of hTERT-transformed NK cells may permit their clinical use. 

A recent review [7] addressed the relationship between female fertility and telomerase activity. The thesis that telomerase activity in the ovaries correlates with general fertility is generally confirmed in the literature, although there are some exceptions. The authors believe that the stimulation of telomerase activity may be a treatment for infertility, although it poses certain risks of activating resting cancer cells. The authors emphasize that proper hygiene and good nutrition are significant factors in maintaining fertility.

Another recent review [8] described the relationship between telomere length and diseases associated with aging: cardiovascular diseases, type 2 diabetes, cancer, Alzheimer’s disease, and osteoporosis. In all these cases, different studies yielded conflicting results. The authors attributed this to the different methods of telomere analysis and the inability to measure telomeres in specific tissues. Instead, in most cases, the telomeres of peripheral blood leukocytes can be measured, which introduces additional difficulties of interpretation.

Another experimental paper [9] established a link between sense and antisense transcription from the TERT promoter. When mutations occur, both processes can be amplified. The authors hypothesized that the increase in antisense transcription is a self-regulatory mechanism that can explain the mutual exclusion of TERT-promoter mutations in vivo.

A very interesting article published by Maugeri et al. [10] describes the effect of pregnancy course on fetal telomere length. When a pregnant woman is overweight or underweight, fetal telomeres become shorter than when the pregnancy occurs in women of a normal weight. This observation highlights the importance of pregnancy itself for the health of the offspring in terms of telomere biology.

The article [11] studied the telomere length and genome instability of primary fibroblasts in idiopathic pulmonary fibrosis (IPF) patients. In addition to telomere shortening, characteristics, such as telomere deletions, micronuclei, anaphase bridges, and dicentrics, were observed in IPF fibroblasts. The changes were more pronounced in patients with detected telomere-related gene mutations compared to IPF patients without these mutations. The authors suggested a more extensive use of karyological analysis for diagnosis procedures.

Nguyen et al. [12] studied the methylation of the hTERT gene. They determined that methylation patterns can predict cell responses to all-trans retinoic acid (ATRA) administration. It is well-known in the literature that ATRA induces hTERT repression in acute promyelocytic leukemia cells, resulting in their differentiation or death. The authors’ envisioned extending the use of ATRA to other cancers based on the methylation of the hTERT gene. 

A review published by Hasanau et al. [13] is devoted to methods of detecting hTERT-promoter mutations for clinical practice. This problem has become so urgent that the WHO has recognized the detection of hTERT-gene-promoter changes as essential for the diagnosis (and hence treatment) of CNS tumors. The methods used, at present, such as Sanger sequencing, droplet digital PCR, next-generation sequencing (NGS), and nanopore sequencing, have their advantages and disadvantages. The differences are related, to various procedures used to obtain tumor material. The authors argued that even a non-invasive diagnosis is possible using new MRI approaches for the analysis of medical image data. 

Our recently conducted review [14] is relevant to understanding the health of the elderly population. The long-known theory of aging related to somatic mutations has received an important medical application. Recently, the phenomenon of CHIP (clonal hematopoiesis of indeterminate potential) was discovered. It is manifested by an abnormally high frequency of certain mutations in hematopoietic lineage cells. One of the previously proposed explanations for this phenomenon is the increased survival rate of cells with proinflammatory changes. Indeed, an analysis of these mutations allows us to notice that they are often accompanied by an increase in proinflammatory characteristics in immune cells, including the key regulators of inflammation, macrophages. We believe that, in this case, a shift towards inflammation in the entire body occurs. Formally, the inflammaging theory connects with the theory of somatic mutations. The consequences are a progressive increase in inflammation alongside age and the comorbidity of various age-associated pathologies. A possible target of intervention could be blood monocytes, namely, affecting their epigenetic memory. 

A review conducted by Rubtsova and Dontsova [15] describes the possible independent role of telomerase RNA transcripts. The noncanonical functions of telomerase components are also discussed.

A review conducted by Lupatov and Yarygin [16] discusses the peculiarities of telomerase regulation occurring in stem cells. The temporal activation of telomerase is capable of altering the stem cells’ proliferative potential. In addition, interesting features of telomere length-regulation activity in the course of embryogenesis are discussed. 

Despite the considerable progress that has been achieved in the field of aging and telomerase research in recent years, several areas still require further research using novel approaches, models, and hypotheses. 

## Figures and Tables

**Figure 1:**
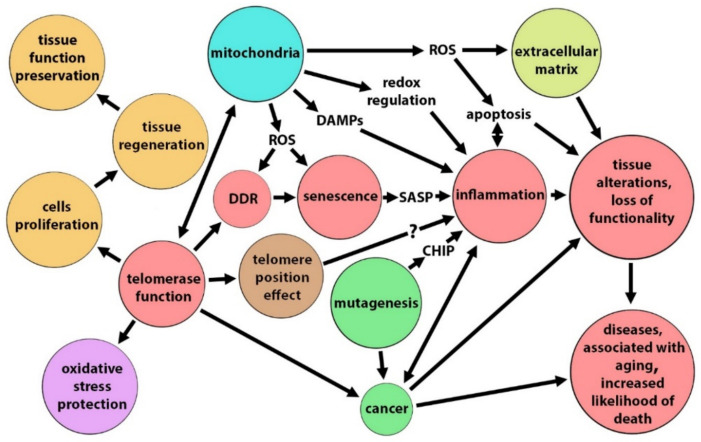
Telomerase involvement in the processes leading to aging or contributing to homeostasis maintenance. ROS—reactive oxygen species, DDR—DNA damage response, DAMPS—damage-associated molecular patterns, SASP—senescence-associated secretory phenotype, CHIP—clonal hematopoiesis of indeterminate potential.
